# Widespread severe myodegeneration in a compound heterozygote female dog with dystrophin deficiency

**DOI:** 10.1002/vms3.433

**Published:** 2021-01-27

**Authors:** Jessica S. Fortin, Chady H. Hakim, Scott Korte, N. Nora Yang, Scott D. Fitzgerald, Gayle C. Johnson, Bruce F. Smith, Dongsheng Duan

**Affiliations:** ^1^ Veterinary Medical Diagnostic Laboratory University of Missouri Columbia MO USA; ^2^ Department of Molecular Microbiology and Immunology School of Medicine University of Missouri Columbia MO USA; ^3^ National Center for Advancing Translational Sciences NIH Bethesda MD USA; ^4^ Office of Animal Resources University of Missouri Columbia MO USA; ^5^ Veterinary Diagnostic Laboratory Michigan State University Lansing MI USA; ^6^ Scott‐Ritchey Research Center College of Veterinary Medicine Auburn University Auburn AL USA; ^7^ Department of Pathobiology College of Veterinary Medicine Auburn University Auburn AL USA; ^8^ Department of Neurology School of Medicine University of Missouri Columbia MO USA; ^9^ Department of Biomedical Sciences College of Veterinary Medicine University of Missouri Columbia MO USA; ^10^ Department of Biomedical Biological & Chemical Engineering College of Engineering University of Missouri Columbia MO USA

**Keywords:** canine, compound heterozygote, Duchenne muscular dystrophy, dystrophin

## Abstract

The University of Missouri (MU) has established a colony of dystrophin‐deficient dogs with a mixed breed background to mirror the variable pathologic effects of dystrophinopathies between persons of a given kindred to further the understanding of the genetic and molecular basis of the variable phenotype; thus to facilitate discovery of an effective therapeutic strategy. Herein we report the phenotype and genotype of a normal‐appearing 10‐month‐old colony female that died suddenly. At necropsy examination, there were reduced skeletal and laryngeal muscle volume and mild dilatation of the oesophagus. Microscopic findings consisted of extensive degeneration and regeneration of the axial skeletal, tongue, oesophageal, and laryngeal muscles that were characterized by considerable central nucleation, individual fibre mineralization and interstitial fibrosis. The myocardial findings were limited to infiltration of adipose cells in the interstitium. The female dog was a compound heterozygote with one X chromosome carrying a point mutation in intron 6 of the dystrophin gene and the other X chromosome carrying a repetitive element insertion in intron 13 of the dystrophin gene. Although the direct cause of death was uncertain, it might likely be due to sudden cardiac death as has been seen in Duchenne muscular dystrophy patients. This case demonstrated dystrophinopathy in female dogs that have no ameliorating normal X chromosome.

## Graphical abstract

A 10‐month‐old mixed breed female dog in a muscular dystrophy research colony at the University of Missouri died suddenly without premonitory symptoms. At necropsy examination, there were reduced skeletal and laryngeal muscle volume and mild dilatation of the esophagus. Microscopic findings consisted of extensive degeneration and regeneration of the axial skeletal, tongue, esophageal, and laryngeal muscles that were characterized by considerable central nucleation, individual fiber mineralization and interstitial fibrosis.

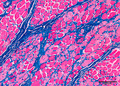

## 1 INTRODUCTION

Muscular dystrophies are heritable degenerative diseases typically identified early in life (Guiraud et al., 2015). The most frequently reported muscle dystrophy occurs because of mutations in the dystrophin gene, a large gene on the X chromosome linked with dystrophin synthesis. Mutations resulting in dystrophin deficiency are denoted as Duchenne muscular dystrophy (DMD) (Guiraud et al., 2015). This muscular dystrophy typically affects males, and females serve as carriers when heterozygous for the mutation. Dystrophin normally localizes at the cytosolic side of the striated muscle sarcolemma protecting the sarcolemma from contraction‐associated shear stress. An absence of dystrophin compromises cell integrity resulting in striated muscle cell injury. DMD occurs at an approximate world‐wide incidence of one in 5,000 human male births (Hoffman et al., 1987; Stark, 2015). However, in DMD males, premature death ensues (Kornegay, 2017). Steroid drugs such as prednisolone and deflazacort, provide symptomatic relief but are not curative, thus DMD represents a disease with poorly met medical need.

Muscular dystrophies have been reported through case reports in numerous breeds of dogs including golden retrievers, Labrador retrievers, Rottweilers, Cavalier King Charles spaniels, French bulldogs, beagles, weimaraners, Samoyeds, miniature schnauzers, old English sheepdogs, Pembroke Welsh corgis and others (Kornegay, 2017; Smith et al., 2011). The most extensively studied muscular dystrophy in veterinary medicine involves dystrophin synthesis deficiency in the golden retriever, with several institutional colonies dedicated to basic research (McGreevy et al., 2015; Shin et al., 2013). In addition, important research colonies also involve Labrador retrievers, beagles and Pembroke Welsh corgis (McGreevy et al., 2015; Smith et al., 2011; Valentine et al., 1988). A driving factor for research funding reflects the surprisingly close homology of the golden retriever muscle dystrophy (GRMD) with Duchenne muscle dystrophy in humans (Smith et al., 2011). An in‐depth comparison of the human, mouse and dog dystrophin‐deficient phenotypes have been published by McGreevy et al., (2015).

Dystrophin gene replacement and repair therapies represent exciting avenues. These types of therapies will not replace the muscle that has already been degraded from significant bouts of degeneration, but will only lessen the severity and progression to a Becker‐like phenotype. Requisite for such approaches will be the identification of the genetic and molecular basis for the DMD phenotype. The most widely used DMD research model is the mdx mouse, but this model does not exhibit good clinical homology to human patients. On the other hand, the canine DMD (cDMD) model shows a phenotype similar to affected boys (Duan, 2011; McGreevy et al., 2015; Nghiem et al., 2017; Smith et al., 2011). Dystrophin gene mutations have been mapped and published in peer reviewed journals in at least nine cDMD dog breeds with point mutations (Cavalier King Charles spaniel muscular dystrophy dogs, golden retriever muscular dystrophy dogs, Rottweiler muscular dystrophy dogs), deletion mutations (Cocker spaniel, Tibetan terrier, German shorthaired pointer), or repetitive element insertions (Pembroke Welsh corgi, Labrador retriever) (Kornegay et al., 2012; Schatzberg et al., 1999; Sharp et al., 1992; Smith et al., 2007, 2011; Walmsley et al., 2010; Winand et al., 1994).

To further the understanding of the genetic and molecular basis for the DMD phenotypes at the University of Missouri, one of the approaches involves outcrossing dogs with dystrophin deficiency in various breeds. The dystrophin mutation of golden retrievers was initially outcrossed onto a Labrador retriever background. Additional outcrossing to other breeds such as Pembroke Welsh corgi and beagle has produced a relatively outbred colony for investigation (Miyazato et al., 2011; Smith et al., 2011). In this quest, a sudden death occurred in a female compound heterozygote. This female was extensively characterized phenotypically and genotypically, as reported herein.

## 2 CASE HISTORY

A 10‐month‐old mixed breed female dog in a muscular dystrophy research colony at the University of Missouri died suddenly without premonitory symptoms. This female was derived through the artificial insemination mating of a carrier female and an affected male. The genotype of the sire is WY. W refers to frame‐shifting mutation in the dystrophin gene due to insertion of a long interspersed repetitive element‐1 (LINE‐1) in intron 13, as reported previously (Smith et al., 2011). The genotype of the dam is XG. G refers to out‐of‐frame mutation in the dystrophin gene due to a point mutation in intron 6 as defined by Cooper et al (Cooper et al., 1988). The creatine kinase levels of the female dog were 322,635 U/L and 84,456 U/L at the age of 1.9 and 6.1 months, respectively.

## 3 RESULTS

At necropsy, axial skeletal muscles were slightly pale and reduced in volume. All skeletal muscles were pale and reduced in volume. Pharyngeal and laryngeal muscles were atrophic. The oesophagus was dilated and flaccid. The lungs were mottled, viscera were congested and the spleen was contracted. Tissue samples were collected into 10% neutral buffered formalin and submitted for processing into paraffin blocks. Tissues in blocks were cut in 5‐micron sections and stained with haematoxylin and eosin (HE). Special stains and immunohistochemistry for dystrophin demonstration were applied on selected tissues.

**FIGURE 1:**
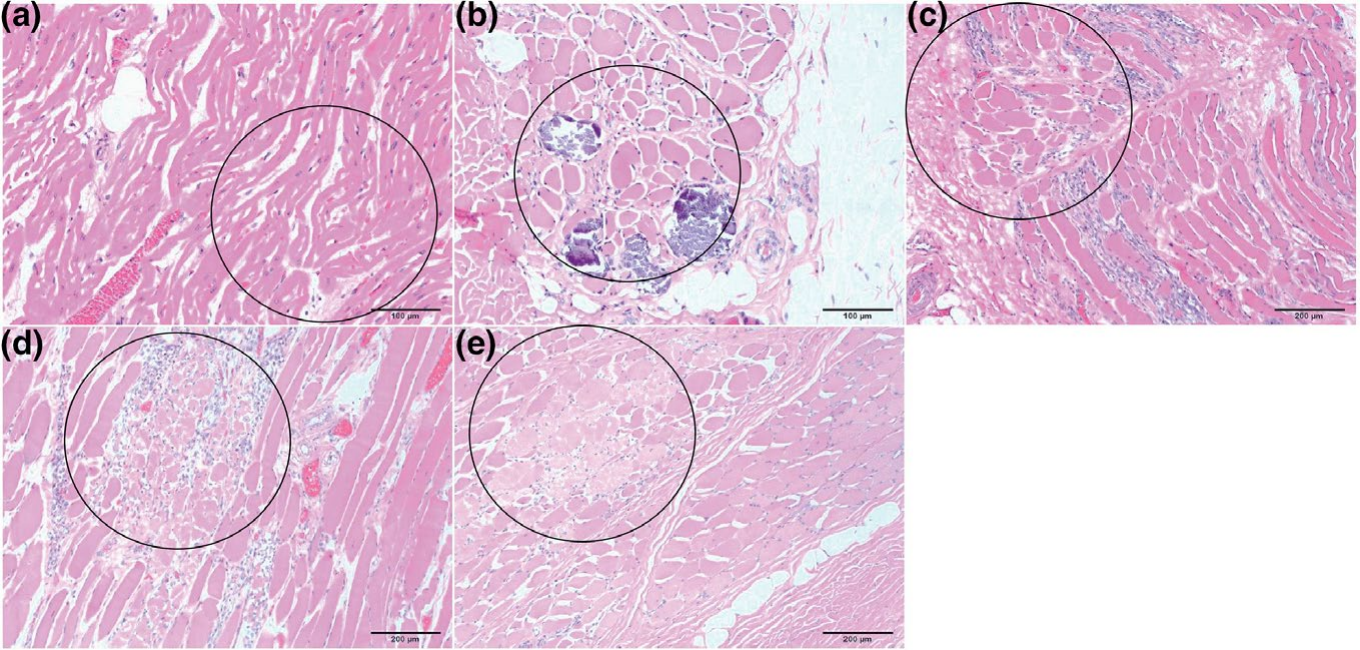
Representative photomicrographs of different muscles of the mixed breed female dog. A representative area for each combination of histopathological changes is encircled. (A) Heart: adipose cells interstitial infiltration and focal area of coarsely branched cardiomyocytes. (B) Oesophageal muscle: multifocal patches of muscle fibrs in cross section have severely diminished diameters with frequent centralized nuclei and nuclear rowing. Mineralization is frequent in such areas. Rare basophilic regenerating fibres are seen. (C) Skeletal muscle of the tongue: similar lesions are found. (D) Skeletal muscle from the front leg: similar pattern of muscular degeneration applies with focal areas of fibre atrophy, central nuclei, slender blue fibres and nuclear rowing. Patches of muscle have also undergone complete coagulative necrosis. There is loss of striations and fibre fragmentation. (E) Skeletal muscle from the rear leg: similar lesions described in the front leg are found. Haematoxylin and eosin stain

**FIGURE 2:**
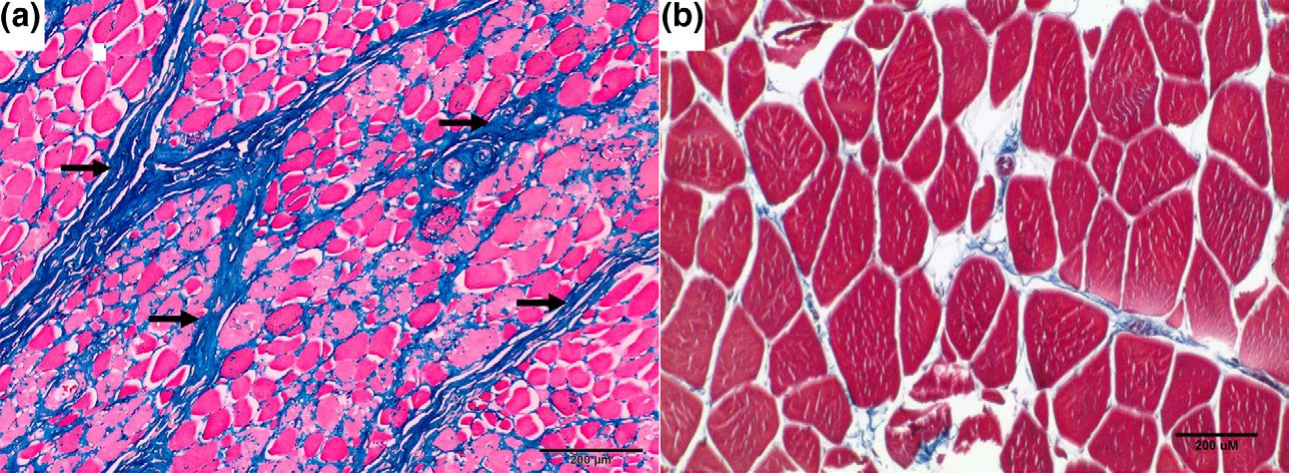
Representative Masson trichrome staining photomicrograph of the skeletal muscle from the leg of the mixed breed female dog. (A) There are marked variability in myofibre size and endomysial fibrosis (arrows). (B) Comparative normal image of mason trichome in an unaffected young dog

**FIGURE 3:**
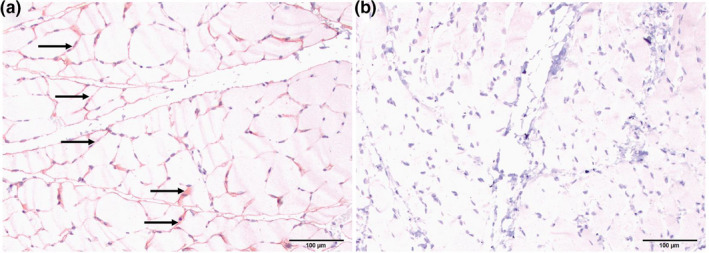
Immunolabelling of dystrophin in axial skeletal muscles of a normal dog (A) and the mixed breed female dog with muscular dystrophy (B). (A) The sarcolemma of a normal dog is labelled immunohistochemically for expression of dystrophin (arrows). (B) The sarcolemmal expression of dystrophin is absent

Sections of the heart (Figure 1a) did not exhibit the severe lesions, as observed in the striated muscles of the tongue, larynx/pharynx, oesophagus and axial skeleton. At these affected sites, striated muscles were altered and characterized by reduced fibre diameter, mature fibrous connective tissue interspersed among individual fibres, centralization and rowing of myofiber nuclei, and patchy areas of fibre mineralization (Figure 1b–e). Loss of fibre striations and patchy areas of coagulation necrosis occurred only in axial skeletal muscles (Figure 1d–e). Fatty infiltration and fibrosis of the myocardium (Figure 1a) and mild pulmonary oedema were noted. Masson's Trichrome confirmed the extension of fibrosis dissecting the myofibre of the heart (Figure 2). The expression of dystrophin in axial skeletal muscles was compared with that of a normal dog by immunohistochemistry. The absence of dystrophin expression was confirmed (Figure 3). Death was attributed to sudden cardiac death, presumably linked with a dysrhythmia associated with the heart lesions.

**FIGURE 4:**
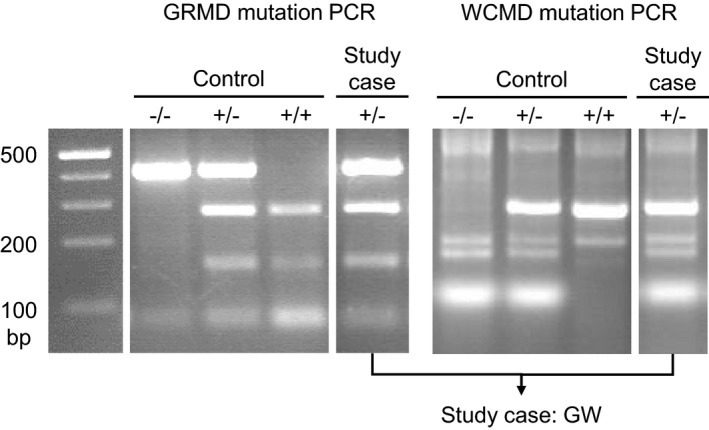
PCR/gel electrophoresis of this study case genotype which exhibits the golden retriever muscular dystrophy (GRMD) and Pembroke Welsh Corgis (WCMD) mutations (GW)

Genotype examination methodology was performed as previously published (Smith et al., 2011) and the PCR/gel is included in Figure 4. The female dog had a splice site point mutation (AGtoGG) in intron 6 of the dystrophin gene (chrX:27926946–27926946) that resulted in aberrant RNA processing. In the other allele, there was a LINE‐1 insertion in intron 13 which resulted in a novel exon with an in‐frame stop codon. The splice site point mutation and LINE‐1 insertion are similar to those found in Golden Retrievers and Pembroke Welsh Corgis with DMD, respectively (Kornegay, 2017; Smith et al., 2011). In this dog, both alleles are mutated at different gene loci, producing a compound heterozygote.

## 4 DISCUSSION

In humans, DMD is an X‐linked recessive mutation in the gene coding for the protein dystrophin. Symptoms develop in boys around the age of two to five years. Most are unable to walk by age 12. The main symptom is voluntary muscle weakness and wasting. Other symptoms include awkward walking, frequent falls, fatigue, poor motor skills, lumbar hyperlordosis and muscle contractures. The average life expectancy is 26 years, with death resulting from cardiorespiratory failure. Well‐managed patients may live into the third or fourth decade of life. Steroid therapy provides symptomatic benefits but is not curative. The mother of the patient may be normal or serve as a carrier. Mothers may show mild symptoms but are often clinically normal. Although 70% of female carriers [people] have histologic abnormalities in skeletal muscle, only 5%–10% manifest mild/moderate correlative symptoms (Dubowitz, 1982). However, sudden death in female carriers has been reported as a consequence of myocardial dysfunctions (Grain et al., 2001; Hoogerwaard et al., 1999; Politano et al., 1996; Schade van Westrum et al., 2011) and histopathological lesions (Kane et al., 2013; Moise et al., 1991; Takano et al., 2011; Yugeta et al., 2006) are present.

The pathologic effects of dystrophinopathies vary between persons of a given kindred; thus the purpose of using cross‐bred animals is to achieve comparable variability in clinical presentations. The University of Missouri has established a colony of dystrophin‐deficient dogs in a mixed breed background to further the understanding of the genetic and molecular basis for the DMD phenotypes, as an approach leading to the development of a gene replacement therapy or CRISPR gene editing therapy. Here we reported the sudden death in a relatively normal‐appearing 10‐month‐old female mixed breed dog from the colony, that had severe and widespread striated muscle myodegeneration characteristic of DMD. Immunohistochemically, we confirmed the lack of dystrophin in muscle. In this case, the female dog had different mutations at different gene loci on each X chromosome. Double mutation indeed happens in humans. Several different cases have been reported. There is one patient with compound mutation (one X chromosome has a deletion of exons 8–13 and the other X chromosome has a splice site mutation (c.10086 + 2T>C) (Soltanzadeh et al., 2010). Another interesting case was reported from a patient who carries the same mutation in both X chromosomes (homozygous for exon 44–45 deletion) (Fujii et al., 2009). Dystrophin labelling of muscle biopsies from heterozygous female carriers of *DMD* mutations who develop symptoms (manifesting carriers) shows a mosaic pattern, i.e. a nonuniform staining of fibres which represent normal and abnormal fibres (Soltanzadeh et al., 2010). A mosaic pattern was not observed in the study case presented herein. It is possible that the antibody used to detect dystrophin in the muscle histological section may not recognize the truncated dystrophin protein.

Clinical laboratory findings in cDMD male dogs are limb weakness and exercise intolerance that appear around 2–3 months of age (Cooper et al., 1988). At approximately 6 months of age, muscle atrophy, joint contracture, hypersalivation, dysphagia, abnormal gait and signs of impaired heart function appear (Bergman et al., 2002; Kornegay, 2017). Death occurs most frequently around 1–3 years of age (McGreevy et al., 2015; Vieira et al., 2015). In contrast to DMD in humans, 20%–30% of cDMD dogs die within 2 weeks of birth. The cDMD dogs also show variation in symptoms. For example, cDMD dogs may be asymptomatic despite absence of dystrophin in striated muscle (McGreevy et al., 2015).

Heterozygous cDMD female dog carriers have been reported to have electrocardiographic abnormalities (Moise et al., 1991; Takano et al., 2011; Yugeta et al., 2006). Sudden deaths have also been reported (Kornegay et al., 1988; Sharp et al., 1992; Valentine et al., 1992). With Holter monitoring, 10 of 11 carriers were reported to exhibit ventricular ectopy, even though baseline electrocardiograms (EKGs) were normal, and each had myocardial lesions identified post mortem (Kane et al., 2013). In controlled experiments effects on growth occur as early as 5 days of age (Smith et al., 2011). In this case, electrocardiography was not assessed. In clinical laboratory assessments of cDMD dogs, creatine kinase levels are consistently elevated whether symptomatic or not (Bergman et al., 2002; Cooper et al., 1988). The creatine kinase levels were elevated in the mixed breed female dog described in this report. The reduction in the CK level at later age (6.1 months versus 1.9 months herein) is due to the loss of muscle. Due to the disease, significant amount of muscle tissue has been replaced by fat or fibrotic tissues. Hence the total CK level was reduced (Hathout et al., 2015).

The consistent alterations found with routine light microscopy preparations of cDMD dog striated muscle include myofibre size variation, central nucleation of myofibres, endomysial fibrosis, multifocal myofibre necrosis, myofibre mineralization, basophilic myofibre regeneration and mononuclear inflammatory cell infiltrates (typically macrophages) (Shiga et al., 2017). These muscle histologic changes are not specific for, but are typical of cDMD dogs. Lesions are severe in males but mild in carrier females, except in the compound heterozygotes, as presented here. Immunohistochemical staining for dystrophin is negative in males and negative in a mosaic pattern in carrier females. IHC for utrophin in sarcolemma is increased in affected dogs (Smith et al., 2011). Utrophin is a membrane protein that compensates for lack of dystrophin (Miyazato et al., 2011). In the heart, fatty infiltration, myofibre hypereosinophilia/fragmentation/vacuolation/shrinkage, myocardial fibrosis, myofiber mineralization, fatty infiltration, and multifocal inflammatory infiltrates are reported to occur. Dystrophin IHC on heart tissues reflects absence in affected males and mosaic areas of positivity in carrier females (Kane et al., 2013; Smith et al., 2011).

Although the cause of death in this animal was not found, we suspect it may very likely be due to ventricular arrhythmia. The female dog that was a compound heterozygote (of the X‐linked dystrophin gene) with widespread myodegeneration. This case demonstrated that more severe pathology can occur in female dogs that have no ameliorating normal X chromosome.

## CONFLICT OF INTEREST

DD is a member of the scientific advisory board for Solid Biosciences LLC and an equity holder of Solid Biosciences LLC. The other authors declare no conflict of interest with respect to the publication of this manuscript.

## AUTHOR CONTRIBUTION

**Jessica S Fortin:** Data curation; Formal analysis; Investigation; Methodology; Validation; Visualization; Writing‐original draft; Writing‐review & editing. **Chady H Hakim:** Data curation; Formal analysis; Investigation; Methodology; Writing‐review & editing. **Scott Korte:** Formal analysis; Validation; Writing‐review & editing. **N. Nora Yang:** Conceptualization; Project administration; Writing‐review & editing. **Scott D. Fitzgerald:** Data curation; Validation; Visualization; Writing‐review & editing. **Gayle C Johnson:** Data curation; Formal analysis; Investigation; Methodology; Supervision; Validation; Visualization; Writing‐review & editing. **Bruce F Smith:** Formal analysis; Validation; Writing‐review & editing. **Dongsheng Duan:** Conceptualization; Data curation; Formal analysis; Funding acquisition; Investigation; Methodology; Project administration; Supervision; Validation; Writing‐review & editing.

## ETHICAL STATEMENT

The authors confirm that the ethical policies of the journal, as noted on the journal's author guidelines page, have been adhered to. No ethics approval was required as this is an investigation of an animal at post‐mortem examination.

### Peer Review

The peer review history for this article is available at https://publons.com/publon/10.1002/vms3.433.
